# Prospective memory and chronic heart failure

**DOI:** 10.1186/1471-2261-13-63

**Published:** 2013-08-28

**Authors:** Tina Habota, Jan Cameron, Skye N McLennan, Chantal F Ski, David R Thompson, Peter G Rendell

**Affiliations:** 1 School of Psychology, Australian Catholic University, Melbourne, Australia; 2 Cardiovascular Research Centre, Australian Catholic University, Melbourne, Australia

**Keywords:** Chronic heart failure, Self-care, Cognitive function, Prospective memory

## Abstract

**Background:**

Patients with chronic heart failure (CHF) experience a number of debilitating symptoms, which impact on activities of daily living and result in poor quality of life. Prospective memory, which is defined as memory to carry out future intentions, has not been investigated in this group. However, emerging evidence suggests CHF patients have difficulties with cognitive processes related to prospective memory. Self-care, which partly relies on prospective memory, is essential in symptom management and preventing acute clinical deterioration. This study aims to measure prospective memory in CHF patients, and examine the relationship between prospective memory and CHF self-care.

**Methods/Design:**

A comprehensive neuropsychological assessment will be conducted to assess a range of cognitive functions and psychopathology. The primary focus will be an assessment of prospective memory using a well-established behavioral measure; Virtual Week. Thirty CHF patients attending a nurse-led management program will be recruited from three hospital sites in Melbourne, Australia and their self-care behaviors will be assessed using the Self-care Chronic Heart Failure Index (SCHFI), a validated self-report tool. An additional 30 healthy controls, matched on age, gender, and IQ will be recruited from the general community.

**Discussion:**

This is a group comparison study that will provide an evaluation of the prospective memory abilities of CHF patients. The findings of this research will provide insight into whether prospective memory may be hindering patients’ ability to perform adequate self-care.

## Background

Patients with chronic heart failure (CHF) represent a rapidly increasing and vulnerable group of individuals with a dismal prognosis [1]. Despite significant improvements in the multidisciplinary management of CHF [2] most patients experience debilitating symptoms that impact on activities of daily living, quality of life, and anxiety and depression, which contribute to increased and frequent hospitalizations, and reduced survival [3]. Due to the poor outcomes associated with CHF, management of this significant chronic condition is a major economic drain on valuable healthcare resources [4]. Multidisciplinary CHF management programs have evolved over recent years to enable the clinical application of evidence-based treatments that reduce economic and patient burden by improving health outcomes such as hospital readmissions [5,6].

A key strategy within CHF management programs is to promote and engage patients to perform specific self-care behaviors [7]. CHF self-care comprises maintenance and management strategies that involve a set of complex cognitive behaviors and decisions [8]. Self-care maintenance includes performing behaviors that help maintain clinical stability, for example medication adherence, and fluid and sodium restriction [8]. Self-care management includes symptom monitoring, for example daily weighing to monitor and recognize changes in symptoms, and then responding to pertinent changes [8]. A case in point is the need to weigh daily and then to respond appropriately to increases in body weight of 2 kg or more. Often, patients do not follow through on this, and despite regular weighing, do not initiate the response needed when there is an increase in weight [9]. Optimal engagement in self-care has the potential to reduce negative health outcomes such as clinical instability, reduce unplanned hospitalizations, and improve survival [10]. Despite the significant research attention towards promoting CHF self-care, many patients have low success in acquiring the necessary skill sets [11]. This is often considered to reflect poor motivation or compliance, due to multiple patient and clinical factors [12,13]. However, the patient’s cognitive ability to respond to vital cues, and initiate appropriate actions, is also critical in predicting engagement in self-care [14].

Cognitive dysfunction has been observed in as many as 75% of patients in select CHF populations [14,15] and it has been linked to changes in cerebral pathology resulting from reduced cerebral perfusion and oxygenation [16]. Consequently, multiple cognitive domains appear to be diminished in CHF patients including language, attention, working memory, visuospatial function, psychomotor speed, and executive function [17,18]. These cognitive deficits may compromise patients’ reasoning and decision-making abilities, thereby limiting their ability to perform self-care [14].

Prospective memory (PM) is defined as memory to carry out future intentions [19]. It involves different phases of forming an intention, holding onto this intention for some time, and then initiating and carrying out the intention at a set time or situation [20]. These phases require the application of multiple cognitive domains including attention, working memory, retrospective memory, and executive functioning [21]. Therefore, prospective memory may also be impaired in CHF patients with significant ramifications in performing self-care tasks. A multiprocess framework has often been used to describe prospective memory functioning. Depending on specific demands of different tasks, or task features, remembering may be either elicited by effortless and automatic processes, or by strategic, attention-demanding processes that include monitoring of the environment for relevant cues [22].

A key distinction between different prospective memory tasks is that some are event-based and others are time-based. Event-based tasks are triggered by an event cue and require monitoring of the environment for that cue [23]. For example, “when I get home in the afternoon [task cue], I have to take my diuretic [PM task]”. On the other hand, time-based tasks are performed at a specific time, or once a specific amount of time has lapsed [23]. This latter type of task requires more strategic monitoring and self-initiated control processes, and consequently results in greater deficits [22]. Another important task distinction is whether a task is regular (same task each day), or irregular (different task each day). Regular tasks impose less demand on retrospective memory (remembering what needs to be done), compared to irregular tasks [24,25].

Failures in prospective memory are often exhibited by individuals with neurological disorders that impact on functional independence [21,26]. Further, a general decline in prospective memory is found in normal aging, particularly after the 60s [27]. Failures in prospective memory have the potential to lead to rapid clinical deterioration in patients with CHF with serious consequences on health outcomes. We propose that prospective memory is crucial for appropriate CHF self-care, for example in tasks involving medication, daily weighing, and initiating an appropriate response to changes in weight.

### Aims

The aims of this study are to determine if: 1) prospective memory ability of CHF patients is impaired compared with an age-matched group, 2) CHF patients exhibit more prospective memory failures in event-based or time-based tasks than matched controls, 3) prospective memory deficits in CHF patients are globalized, or specific to a particular type of prospective memory task, 4) prospective memory ability correlates with self-care maintenance behaviors, management skills, and confidence, and 5) prospective memory ability correlates to functioning as assessed by the Heart Failure Screening Tool (Heart-FaST). Findings from this study will contribute to our understanding of the factors that predict adequate engagement in CHF self-care and provide avenues for developing appropriate interventions.

## Methods/Design

This study will use a group comparison design to examine the prospective memory abilities of patients with CHF. Groups will be matched on age, gender, years of education, and premorbid intelligence estimated/indexed by the National Adult Reading Test (NART) [28].

### Participants

Participants will include adults 18 years and over, although due to the prevalence of CHF in the elderly, the majority of the sample will be above 50 years. The CHF group will have a confirmed diagnosis of CHF based on national guidelines [29]. All participants will be recruited from a nurse-led CHF management program at one of three public hospitals in Metropolitan Melbourne, Australia. Patients will be excluded if they reside in a residential aged high care facility, have a documented history of moderate-to-severe cognitive impairment or dementia (based on the Addenbrooke’s Cognitive Examination-Revised; ACE-R) [30] or have a terminal diagnosis. Participants who do not have sufficient comprehension to read English without the need of a translator will be excluded. The control group will be recruited through flyer advertising in the general community, and snowball recruitment. Participants being considered for the control group will be excluded if they have a history of CHF or neurological disease, and/or have had recent treatment (past three months) for an acute cardiovascular problem.

#### CHF participant descriptive data

Information relating to a range of participant variables will be collected in order to characterize the sample. These variables include demographic details, number and quality of social supports, education level, occupation status and history, history and current treatment of depression and/or anxiety, and comorbid illness burden (measured using the Charlson Comorbidity Index). Cardiac related history will also be obtained, including cardiovascular risk factors, length of time living with chronic heart failure, type of chronic heart failure diagnosis, chronic heart failure etiology, prior treatments for chronic heart failure (beyond medical therapy), observations of clinical features, and self-care behaviors (measured using the Self-care Chronic Heart Failure Index v6, (SCHFI) [31] and ability (measured using Heart-FaST) [32].

The SCHFI is a comprehensive self-report instrument of self-care practices pertinent to the management of CHF. The SCHFI comprises 15 items rated on a 4-point response scale. It has three subscales: maintenance, management and confidence [31]. Self-care maintenance items assess treatment adherence and symptom monitoring to prevent clinical deterioration, for example fluid restrictions and daily weighing. Self-care management items assess the ability to recognize changes in CHF symptoms, evaluate the significance of the changes, and make decisions on treatment actions, for example in the event of >2kg weight gain a treatment action would be to take an extra diuretic. Self-care confidence items measure perceived ability to engage in each self-care phase and help to explain why some patients master self-care and others do not [33]. Scores from each of the three self-care scales are transformed to 100-point scales; higher scores reflect better self-care. Self-care management scores are only computed for those patients reporting CHF symptoms of ankle swelling or trouble breathing in the previous three months [31]. Scale scores >70 are considered to indicate adequate self-care [31]. The SCHFI is a reliable measure of self-reported self-care skills and behaviors and has been extensively validated among CHF populations around the world [34].

The Heart-FaST was developed to assist clinicians in applying educational and support strategies based on self-care capacity [32]. Unlike the SCHFI, which primarily assesses self-care practices, the Heart-FaST is a unique instrument that assesses identified barriers in patient engagement in self-care behaviors. The construction of the Heart-FaST was based on the results of extensive literature review, development and testing of the InCOGNITO conceptual model [12] and expert opinion in carefully selecting relevant items. The Heart-FaST comprises three salient domains recognized as barriers to CHF self-care: cognitive, emotional and physical functioning. Possible scores on the three Heart-FaST domains are: 0 to 3 on physical functioning, 0 to 20 on cognitive functioning, and 7 to 49 on emotional functioning. Lower scores indicate higher functioning on each domain and better self-care capacity [32]. Levels of functioning across each Heart-FaST domain are graded as low, medium or high. Nursing recommendations and guidelines directed at applying individual educational and support strategies for each level of functioning have been developed. Initial pilot data indicates that the Heart-FaST is a valid instrument for assessing self-care capacity in patients with CHF and is likely to aid nurses in tailoring support strategies to promote effective CHF self-care [35].

### Cognitive measures

#### Global cognition

The *Addenbrooke’s Cognitive Examination–Revised* is a short, sensitive cognitive screening test that measures five cognitive domains; attention/orientation, memory, verbal fluency, language and visuospatial abilities. Lower scores suggest poorer cognitive performance [30]. The ACE-R is sensitive to early stages of dementia [30] and will be used to identify and exclude potential participants who have moderate-to-severe cognitive impairment.

#### Primary measure

**Figure 1:**
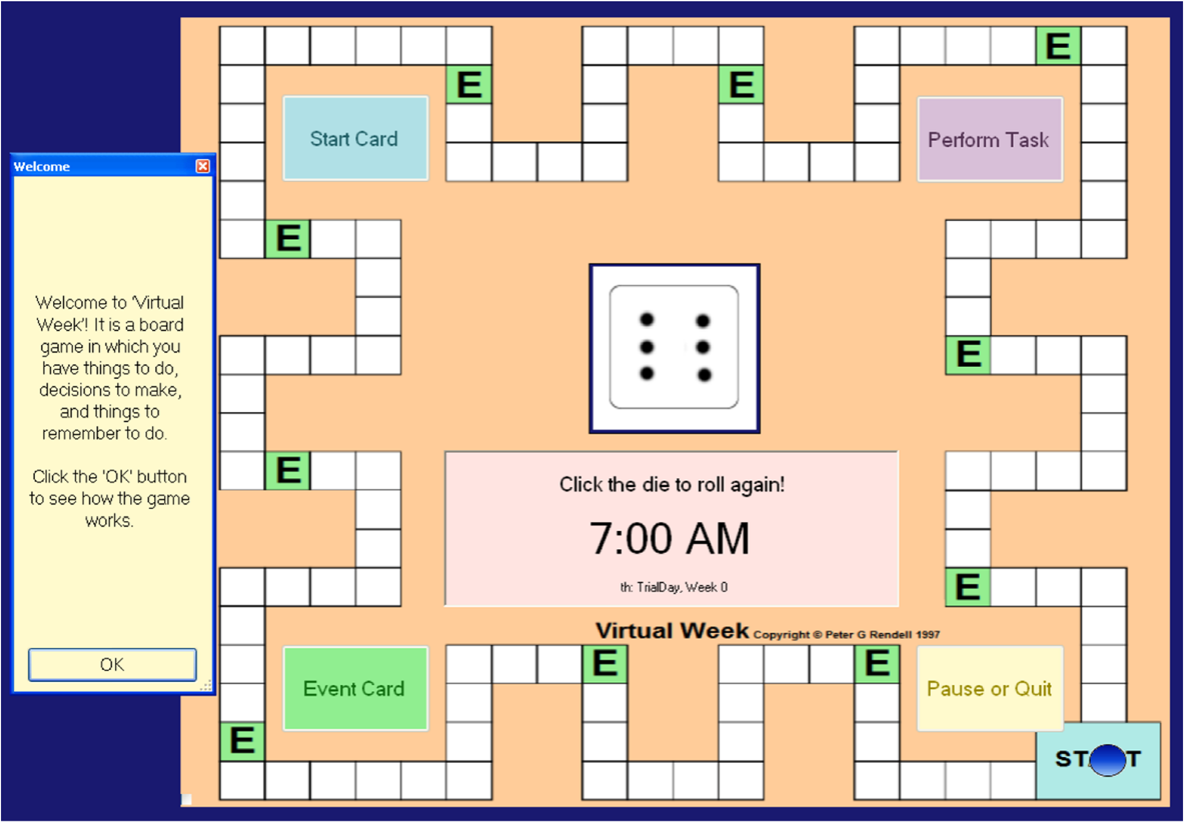
Virtual Week.

Prospective memory will be the primary focus of this research project and will be measured using the well-established behavioral measure, *Virtual Week;* a computerized board game that stimulates a week of everyday activities (Figure 1). The study will use a shortened two-day version of the game. A key advantage of Virtual Week is that it allows tasks with different task features (event, time, regular, irregular) that closely represent activities in real life, to be investigated systematically in a controlled manner. Virtual Week has been used widely within prospective memory research and has demonstrated robust psychometric properties [19,36]. A study using a group of participants with schizophrenia found the split-half reliability estimate to be 0.90 for the overall measure [37]. In another study using a clinical sample, the reliability estimate was reported to be 0.89 for Parkinson’s Disease patients and 0.81 for the control group [25]. It has been successfully used in previous research within clinical and normal populations [19], including several studies with abnormal ageing [26], and it is an interesting and intuitive task for users which are important features for maintaining motivation for task completion.

**Figure 2:**
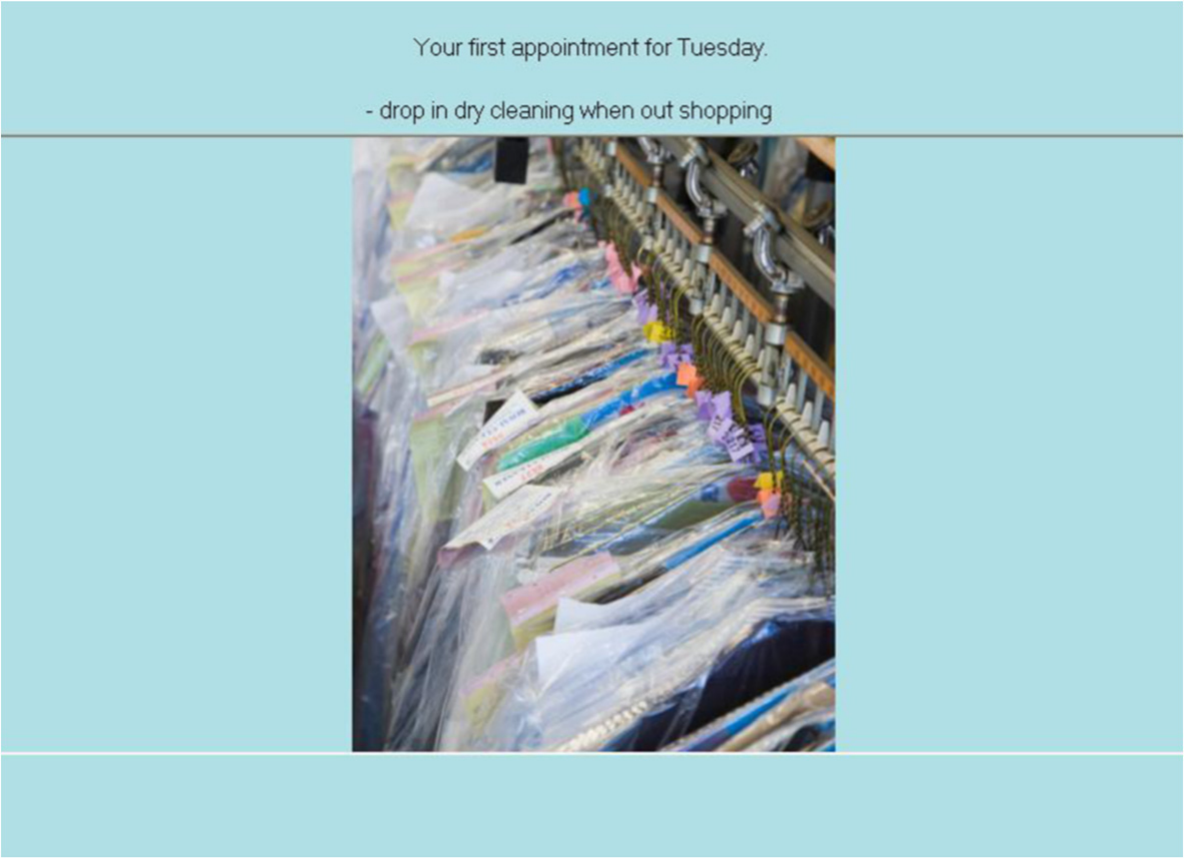
Prospective memory task.

**Figure 3:**
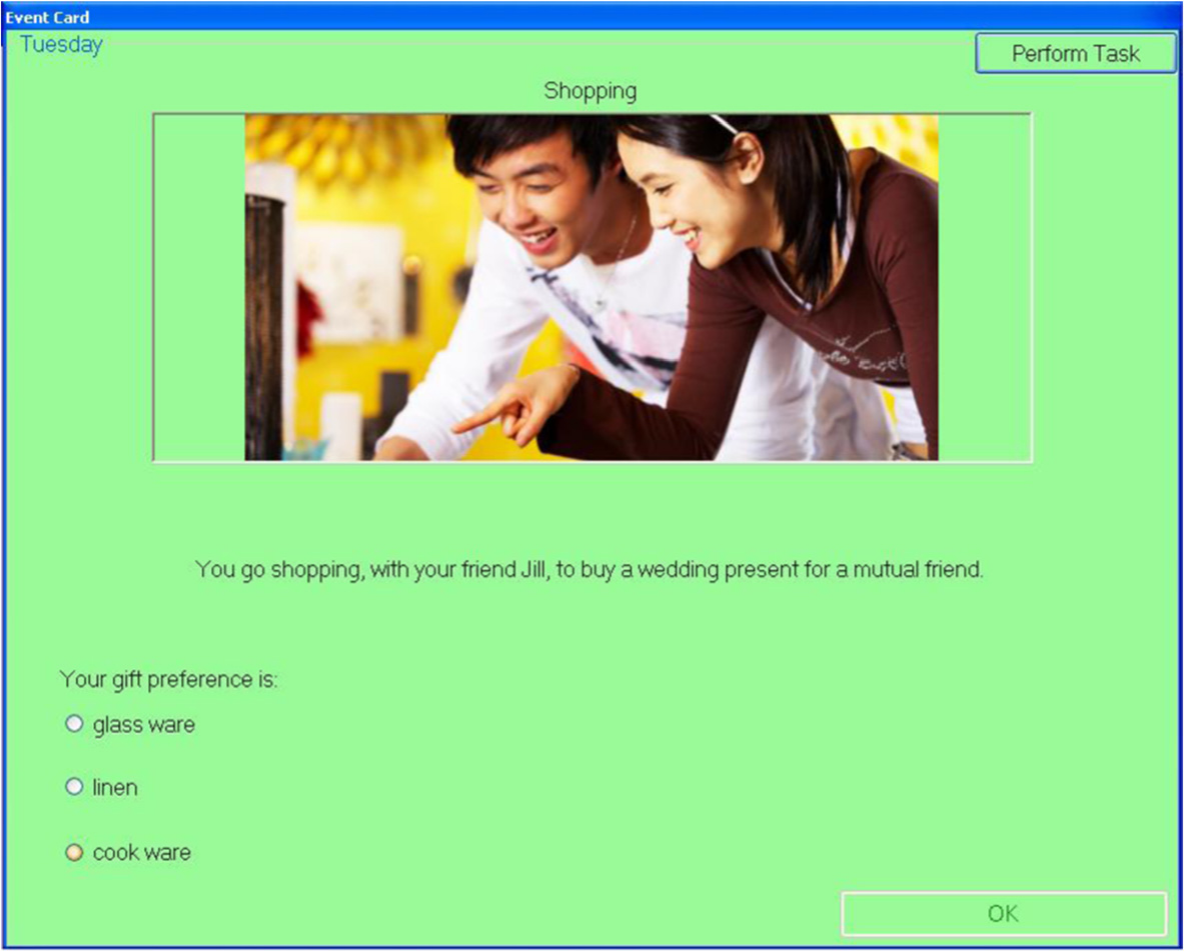
Event card.

**Figure 4:**
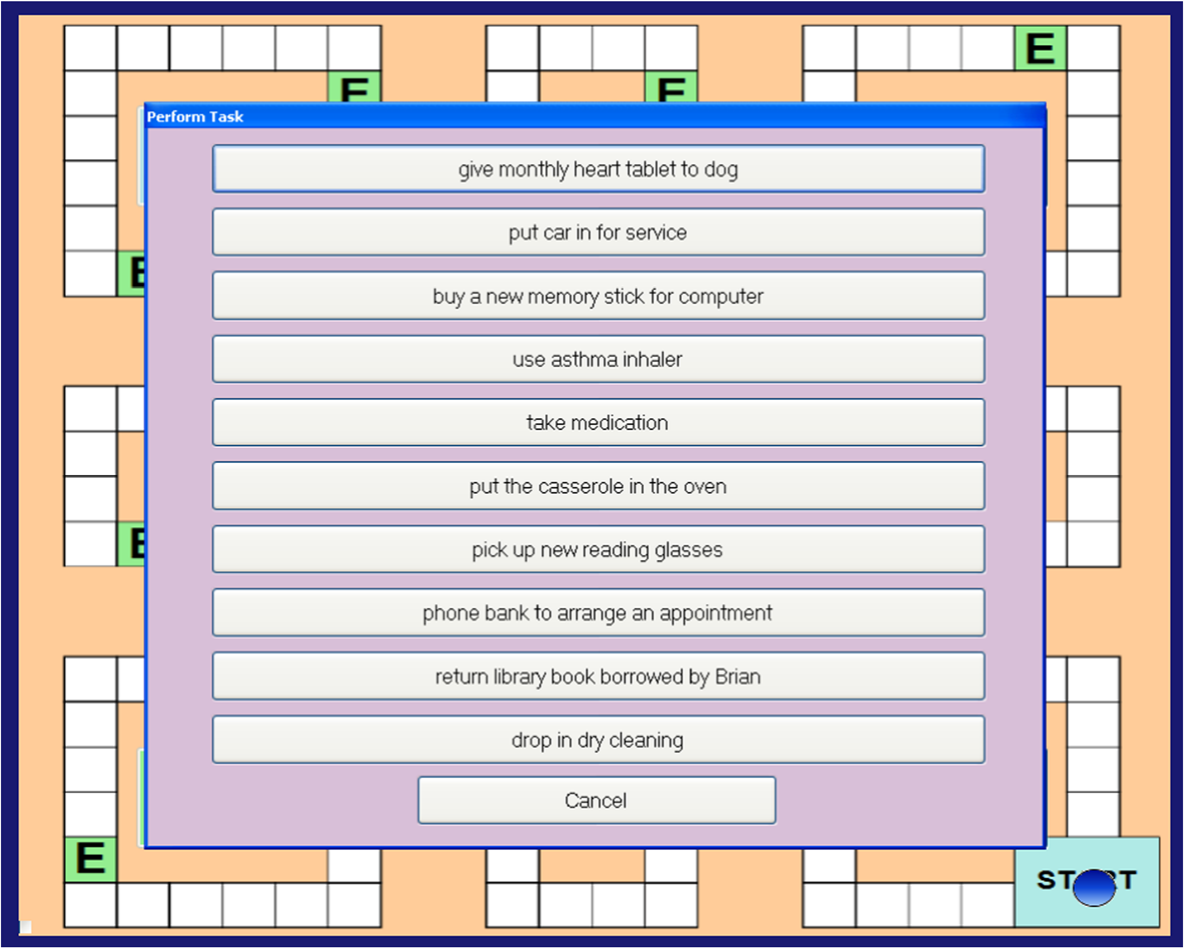
Prospective memory task execution.

Participants will move a token around the board game on the roll of a dice, with each circuit of the board representing one virtual day. As participants move around the board, a series of events occur (e.g., *‘you go to the library’*) where the participant is required to make choices about the tasks relevant to the event (e.g., mode of transport to library). At some of these events, participants have to remember to perform a prospective memory task. For example, at the beginning of the game, participants might be asked to remember to ‘drop in the dry cleaning’ (PM task) when ‘shopping’ (irregular, event-based task; Figure 2). As they move around the board, they will be instructed at several points during the day, to pick up an ‘event card’ when they land on, or go past, an ‘event’ square (represented by an ‘E’). If the event card is ‘shopping’ (Figure 3), participants should action the prospective memory task by clicking on a ‘perform task’ button and selecting the prospective memory task that needs to be performed (Figure 4). Other tasks will have to be performed at a specified time of day. For example, participants will be asked to attend a meeting with a librarian at 3pm (irregular, time-based task). They will be required to monitor a virtual clock in the game (calibrated to position of token on board) and perform the task by clicking on the ‘perform task’ button at the specified time period. The perform task button reveals a list of possible tasks for participants to select. Participants will also be asked to take medication at the ‘breakfast’ and ‘dinner’ event cards each day (regular, event-based task), and take their asthma inhaler at two time points during the day (regular, time-based task).

### Characterization measures

#### Premorbid intelligence

The *National Adult Reading Test*[28] will be used as an index of premorbid intelligence. This is a word-recognition test of vocabulary knowledge that requires participants to read aloud 50 English words of increasing difficulty that do not follow normal phonetic rules, for example, ‘chord’. Scores on the NART correlate with general IQ, including Verbal IQ and Perceptual IQ [38]. On the basis of number of errors in pronunciation, a Full-Scale IQ estimate of the Wechsler Adult Intelligence Scale-Revised can be derived. The NART is a frequently used test that is a valid and reliable measure. It has high construct validly [39], and internal reliability estimates are reported to be above 0.90 [40].

#### Psychopathology

The *Hospital Anxiety and Depression Scale* (HADS) [41] is a 14 item questionnaire, which will be used to asses psychopathology. Seven of the items relate to anxiety and seven relate to depression. Responses are provided on a Likert scale, and scores on each scale are interpreted in ranges: normal (0-7), mild (8-10), moderate (11-14), and severe (15-21). The two subscales have a mean correlation of 0.56, and the mean Cronbach’s alpha is 0.83 for anxiety and 0.82 for depression [42]. The HADS has been used successfully in psychiatric patients and in the general population [42].

#### Executive functioning

Four measures will be used to assess participants’ executive functioning. Executive functions are higher order thinking processes.

The *Trail Making Test* (TMT) [43] assesses planning ability and divided attention. It is a pencil and paper test and consists of two parts. In Part A, participants are required to draw lines to connect circles that are numbered consecutively; in Part B, participants must connect circles that are numbered and lettered, alternating between the numeric and alphabetic sequences. The total time taken to complete the task is measured. Faster performance on the TMT indicates higher levels of planning ability. The TMT has successfully been used within normal and clinical groups [38], and the reliability for neurologically stable groups is reported to be at least 0.70 for Part A and Part B [44].

The *Hayling Sentence Completion Test*[45] assesses cognitive initiation and inhibition. First, participants verbally complete 15 sentences with an obvious response. For example, when presented with the sentence “The captain wanted to stay with the sinking …”, they must provide the word ‘ship’. In the second part, participants have to suppress the obvious response and complete 15 sentences with an unrelated word. The participant’s response times for each section, and their errors, determine their overall score. High split-half reliability coefficients have been reported for a brain impaired sample (Hayling 1 time, 0.93, Hayling 2 time, 0.80, and Hayling errors, 0.72), but reliability is more varied (0.35 to 0.83) for healthy adults [45].

The *Digit Span*, a subtest of the Wechsler Adult Intelligence Scale-IV (WAIS-IV) [46] is a measure of working memory, which is the ability to mentally hold and manipulate new information within a limited time frame. Participants are verbally presented with a string of numbers (e.g., 7-2-8-6) and they are required to remember and repeat these numbers in a specific order, either forwards (e.g., 7-2-8-6), backwards (e.g., 6-8-2-7), or in sequence (lowest to highest, e.g., 2-6-7-8). Participants are scored out of 16 for each section of the test. Reliability coefficients for the WAIS-IV Digit Span are all reported to be above 0.90 [46].

The final measure of executive functioning is an adaptation of the Controlled Oral Word Association task, which is a measure of *verbal fluency*[47]. Two types of verbal fluency will be assessed, phonemic and categorical. In the phonemic verbal fluency task, participants are required to orally generate as many words as they can beginning with the letters P, R and W, excluding proper nouns, numbers, and repetitions of the same word with a different suffix. Participants are then required to name as many animals as they can, beginning with any letter, as a measure of their semantic verbal fluency. Participants are given one minute for each task. Although other letter combinations have been used previously, differences between versions appear to be negligible; correlations of 0.82, or higher, have been reported for two sets of letters (e.g., PRW, CFL) [38,47].

#### Verbal memory

The *Rey Auditory Verbal Learning Test* (RAVLT) [48] will be used to measure verbal memory and provides a measure of immediate recall, delayed recall and recognition. This test involves a list of 15 words, which an examiner reads aloud. The participant’s task is to repeat all the words they can remember, in any order. This procedure is carried out a total of five times. After a 20-minute delay period filled with other activities, the participant is asked to recall as many words as possible. Finally, a recognition test is administered. Participants are presented with a list of 30 words (15 distracter items, and the 15 list words), and are asked to identify as many of the list words as possible. The RAVLT is a recognized measure of memory that is widely used in research as well as in clinical practice [38]. The RAVLT has a high internal reliability of 0.90 for the total score [49].

### Procedure

**Figure 5:**
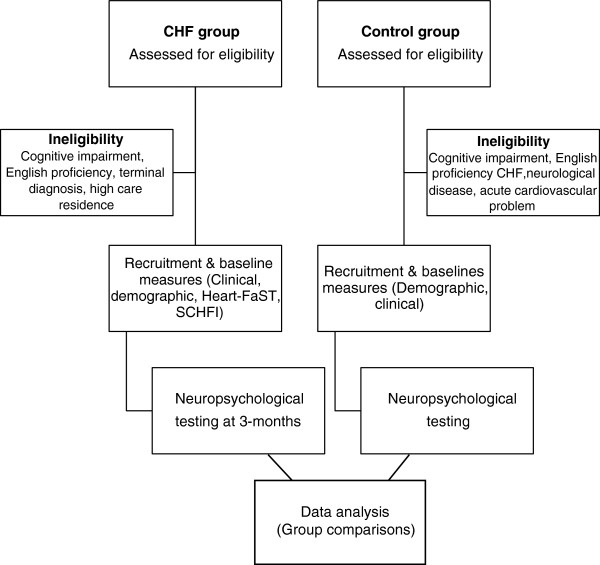
Study protocol.

A research assistant, in collaboration with the CHF nurses, will screen and recruit participants for the CHF group. A detailed history will be collated based on patient self-report information and review of medical records. Around three months later, participants will be tested in a single session, lasting between two to three hours. The delay of three months will be built in to ensure that participants are medically stable when they complete the neuropsychological assessment. The ACE-R will be administered first to assess each participant’s cognitive functioning. Virtual Week will be administered next, followed by the remaining measures. Participants will be offered at least one break throughout the session and will be encouraged to take additional breaks as needed. The study protocol is illustrated in Figure 5. Participant inclusion and exclusion criteria have been described above.

### Data analyses and power calculation

A descriptive analysis of all variables will be performed. A mixed factorial ANOVA will be used to examine main effects and interactions of prospective memory tasks, across CHF patients and healthy controls. Statistical significance will be considered at a *p*-value of <0.05. As participants will be required to perform different prospective memory tasks (event-based, time-based, regular, irregular), analyses will also examine whether prospective memory lapses are specific to a particular task, or pervasive across tasks. Comparisons based on normative samples will also be reported for the cognitive screen (ACE-R), measure of premorbid intelligence (NART), three measures of executive function (TMT, Digit Span, Hayling), and verbal memory (RAVLT; Trials 1-5).

Sample size estimations are based on a previous study using Virtual Week with a clinical sample involving multiple sclerosis (MS) patients [50]. Almost half of patients with MS develop neurocognitive dysfunction in the areas of memory, attention, concentration, and executive function [51]. The cognitive deficits in patients with MS are modest compared to CHF patients, therefore the following power calculation is a conservative estimate–based on the magnitude of difference in prospective memory performance between MS patients and matched controls [50], to get an effect size of 0.84, a sample of 60 (30 in each group) will produce a power of 0.89.

Exploratory correlation analyses will be conducted to examine the relationship between different prospective memory types (event, time, regular, irregular) and the three domains of self-care: self-care maintenance behaviors; management skills; and confidence. Further exploratory correlations will investigate the relationship between the three clinical factors (cognitive, emotional and physical functioning) assessed by the Heart-FaST, and self-care ability. Preliminary correlations will also be conducted to examine the association between Heart-FaST and the behavioral measure of prospective memory, Virtual Week.

### Ethics

This study has been approved by the Eastern Health Research and Ethics Committee (LR39/1112), and the Human Research Ethics Committee of the Australian Catholic University (V2007 08 69; 2012 O4V). Informed consent will be obtained from all the participants in the study.

## Discussion

The results of this study will be the first to provide insights into the prospective memory difficulties experienced by patients with CHF. The study will test the hypothesis that prospective memory abilities are impaired, compared to healthy controls. Prospective memory failures experienced by CHF patients could affect self-care, for example by forgetting to pick up prescription medications from the pharmacist, forgetting to attend doctor appointments, forgetting to take daily medications and performing daily weighing, and failing to respond to >2kg changes in weight, all of which are considered crucial for the management of their condition [7]. Therefore, this study will also test the hypothesis that prospective memory is a significant predictor of a patient’s ability to engage in self-care behaviors and respond appropriately when sudden changes occur. If the findings indicate that prospective memory is associated with poor self-care outcomes, it will provide directions for research addressing cognitive impairments experienced by CHF patients. The findings also have the potential to provide avenues for implementing individually tailored patient education and support strategies, dependent upon individual capabilities identified through screening.

A fundamental approach to improving adverse CHF patient outcomes and associated healthcare costs lies in applying individualized education and support strategies [52]. Improving prospective memory alongside the individualized application of educational and support strategies is likely to enhance critical everyday self-care actions and decisions. Combining these two complimentary approaches into CHF management programs will increase the likelihood of enhancing functional independence for individuals diagnosed with CHF.

A number of practical considerations in undertaking this study have been considered and addressed. Given that CHF patients are typically older adults, a touch screen will be made available for participants who are either unwilling or unable to use a computer and mouse for testing. Using a simulated touch screen with participants pointing on screen and experimenters operating mouse overcame previous reluctance or difficulties of adults using a computer when playing Virtual Week [53]. We acknowledge that the length of neuropsychological testing may cause fatigue, particularly in the CHF group. Therefore, participants will be provided breaks as necessary. Alternatively, testing will be divided across two sessions within a one-week period in order to avoid significant cognitive fluctuations between sessions. All CHF participants will be tested at approximately three months following enrolment into a CHF management program; this will ensure that patients are tested during a period when they are more likely to be clinically stable.

## Abbreviations

ACE-R: Addenbrooke’s Cognitive Examination-Revised; CHF: Chronic heart failure; HADS: Hospital Anxiety and Depression Scale; Heart-FaST: Heart-Failure Screening Tool; MS: Multiple sclerosis; NART: National Adult Reading Test; PM: Prospective memory; RAVLT: Rey Auditory Verbal Learning Test; SCHFI: Self-care Chronic Heart Failure Index; TMT: Trail Making Test; WAIS-IV: Wechsler Adult Intelligence Scale-IV.

## Competing interests

The authors declare that they have no competing interests.

## Authors’ contributions

Principal responsibility for study design and conduct is assumed by JC and PGR. TH is involved in the data collection and overall project management; all other authors are responsible for the supervision of the project and its ethical approval and conduct. TH, JC, and SNM drafted the manuscript. All authors provided critical review for important intellectual content and approved the final manuscript.

## Authors’ information

^1^TH is a registered psychologist who is currently undertaking her Doctor of Philosophy and Masters of Clinical Psychology. ^2^JC is a Senior Research Fellow (Cardiovascular Research Centre) with a program of research aimed at developing screening methods and informing clinical practice in the application of individualized patient education and management strategies directed at improving health outcomes among patients diagnosed with heart failure. ^1^SNM is a lecturer in the School of Psychology, and she has previously worked as a practicing psychologist and research psychologist. ^2^CFS is an Associate Professor of Research in the Cardiovascular Research Centre where she co-heads the psychosocial program of research. ^2^DRT is a Professor of Nursing in the Cardiovascular Research Centre where he heads the psychosocial program of research. ^1^PGR is a Professor in the School of Psychology. He is currently leading the Cognition and Emotion Research Lab that conducts experimental psychology research in the field of cognitive psychology and neuropsychology.

## Pre-publication history

The pre-publication history for this paper can be accessed here:

http://www.biomedcentral.com/1471-2261/13/63/prepub
